# Tyrosines involved in the activity of φ29 single-stranded DNA binding protein

**DOI:** 10.1371/journal.pone.0217248

**Published:** 2019-05-20

**Authors:** Iván de la Torre, Victor Quiñones, Margarita Salas, Alicia del Prado

**Affiliations:** Centro de Biología Molecular “Severo Ochoa,” (Consejo Superior de Investigaciones Científicas-Universidad Autónoma de Madrid), Universidad Autónoma, Cantoblanco, Madrid, Spain; Centre National de la Recherche Scientifique, FRANCE

## Abstract

The genome of *Bacillus subtilis* phage ϕ29 consists of a linear double-stranded DNA with a terminal protein (TP) covalently linked to each 5’ end (TP-DNA). ϕ29 DNA polymerase is the enzyme responsible for viral DNA replication, due to its distinctive properties: high processivity and strand displacement capacity, being able to replicate the entire genome without requiring the assistance of processivity or unwinding factors, unlike most replicases. ϕ29 single-stranded DNA binding protein (SSB) is encoded by the viral gene 5 and binds the ssDNA generated in the replication of the ϕ29 TP-DNA. It has been described to stimulate the DNA elongation rate during the DNA replication. Previous studies proposed residues Tyr50, Tyr57 and Tyr76 as ligands of ssDNA. The role of two of these residues has been determined in this work by site-directed mutagenesis. Our results showed that mutant derivative Y57A was unable to bind to ssDNA, to stimulate the DNA elongation and to displace oligonucleotides annealed to M13 ssDNA, whereas mutant Y50A behaved like the wild-type SSB.

## Introduction

Bacteriophage ϕ29 is a lytic phage that infects *Bacillus subtilis*. Its genome consists of a linear double stranded DNA (dsDNA) molecule of 19,285 base pairs (bp) with a terminal protein (TP) covalently linked to each 5’ DNA end (parental TP). The minimal replication origin is formed by the parental TP together with the terminal 12 bp containing a 6 bp inverted terminal repeat (3’-TTTCAT) [1]. The ϕ29 DNA polymerase forms a heterodimer with a free TP molecule (primer TP) that recognizes the replication origins. The ϕ29 double-stranded DNA binding protein (DBP) binds all along ϕ29 DNA, forming a nucleoprotein complex at the replication origins that has been proposed to open the DNA ends, facilitating the initiation step of replication [2]. Once the replication origin is recognized by the heterodimer, the ϕ29 DNA polymerase catalyses the formation of a phosphoester bond between the initiating dAMP and the hydroxyl group of Ser232 of the TP [3]. The initiation occurs opposite the 3’ second nucleotide of the template strand. This TP-dAMP complex is translocated backwards one position to recover the template information corresponding to the first 3’-T [4]. There is a transition stage between the initiation with TP and the elongation with the DNA. When the polymerase reaches the tenth nucleotide, it is dissociated from the TP [5] and the same polymerase catalyses processive chain elongation of the complete ϕ29 genome via a strand displacement mechanism [6].

Replication starts at both ϕ29 DNA ends generating the type I replication intermediates (RI), consisting of full-length dsDNA molecules with two branches of single-stranded DNA (ssDNA) that are stretched by the single-stranded DNA binding protein [7–9]. When the two replicative forks converge, a type I RI becomes physically separated into two type II RI [10].

SSBs are present through all kingdoms of life and although they have similar functions, their structures are different (reviewed in [11]. These proteins are indispensable elements which play essential roles in DNA replication, recombination and repair in bacteria, archaea and eukarya as well as in viruses and other genetic mobile elements [12], avoiding the formation of secondary structures or the attack by nucleases (reviewed in [11, 12]. From the structural point of view, SSBs usually bind to ssDNA in a non-specific way through a structural domain named oligonucleotide/oligosaccharide binding-fold (OB-fold). This domain is usually formed of five-stranded β barrel and an α-helix between strands 3 and 4 (reviewed in [13]) and interacts with the ssDNA by non-specific base-stacking with aromatic residues and also by electrostatic interactions (reviewed in [11]). These domains are capable of binding a variety of ligands, in addition to the ssDNA (reviewed in [14]). The structure of some SSBs, from bacteriophages as RB69 [15] to humans [16], has been obtained, and even some of them have been recently characterized in complex with ssDNA as in the case of *Enterobacter cancerogenus* bacteriophage Enc34 [17] or *E*.*coli* SSB [18].

The ϕ29 SSB is encoded by the viral gene 5. It has 124 amino acids and a molecular weight of 13 kDa. It protects DNA from nucleases [19] and prevents unproductive binding of ϕ29 DNA polymerase to ssDNA [8]. *In vivo*, ϕ29 SSB seems to play an essential role in DNA replication [20], although recent studies suggested that ϕ29 SSB could be dispensable for viral replication in a temperature dependent fashion [21]. ϕ29 SSB stimulates the dNMP incorporation rate by ϕ29 DNA polymerase during replication of TP-DNA or primed M13 ssDNA [22]. In addition, ϕ29 SSB also has a helix-destabilizing activity or DNA unwinding activity, which would help ϕ29 DNA polymerase in strand displacement DNA replication when DNA opening is impaired [22, 23]. The helix-destabilizing activity carried out in type I RI [22], together with its dynamic dissociation from the DNA ahead the polymerase in type II RI, make ϕ29 SSB really efficient in the stimulation of viral DNA replication [22, 24]. The progression of ϕ29 DNA replication into type II replication intermediates implies that the complex between ssDNA and ϕ29 SSB ahead of the DNA polymerase has to dissociate in order to convert ssDNA into dsDNA, making the binding to ssDNA highly dynamic with a high dissociation rate. ϕ29 SSB is monomeric in solution and shows a low DNA-protein complex stability [24]. Each ϕ29 SSB has a moderate cooperative binding to 3–4 nucleotides of ϕ29 TP-DNA, being required in stoichiometric quantities with respect to the substrate [24]. Consequently, ϕ29 SSB is extremely abundant in the infected *B*. *subtilis* [19] and it is required in high amounts for *in vitro* amplification [25]. The residues Tyr50, Tyr57 and Tyr76 were suggested by intrinsic tyrosine fluorescence quenching assays to be involved in binding the ssDNA [26, 27].

The objective of this paper was to study the role of ϕ29 SSB residues Tyr50, Tyr57 and Tyr76 in the interaction with ssDNA and its involvement in ϕ29 TP-DNA replication by functional analyses of the mutant derivatives Y50A, Y57A and Y76A. The experiments were carried out with Y50A and Y57A because variant Y76A was insoluble and could not be purified. The results presented in this work showed the importance of residue Tyr57 in binding to ssDNA, in the stimulation of DNA elongation and in the unwinding activity.

## Materials and methods

### Nucleotides and DNAs

Unlabeled nucleotides were supplied by GE Healthcare. The [α-^32^P]dATP (3,000 Ci/mmol) and [γ-^32^P]ATP (3,000 Ci/mmol) were obtained from PerkinElmer.

The ϕ29 TP-DNA was isolated as described [28] and M13mp18 ssDNA was from the laboratory stock (protocol for purification included in S2 Text). The sequence of the oligonucleotides used was: 17mer supplied by SIGMA (5’ GTTTTCCCAGTCACGAC). The oligonucleotide was 5’-labeled with [γ-^32^P]ATP and T4 polynucleotide kinase for the unwinding assay.

### Proteins

The ϕ29 DNA polymerase wild-type and the exonuclease-deficient mutant D12A/D66A [29] were purified as described [30]. The ϕ29 terminal protein (TP) and ϕ29 double stranded DNA binding protein (DBP) were purified as described [31]. T4 polynucleotide kinase was purchased from New England Biolabs. The ϕ29 SSB wild-type and mutants were purified as explained in S1 Text (S1 and S2 Figs show the induction and purification of the SSBs and the purified proteins, respectively).

### Gel mobility shift assay (EMSA)

A fragment of the gene yshC of *B*. *subtilis* was amplified by using the oligonucleotides: 5’ CCGCGGATCCCATCATTTTACGGG and 5’ GGGTCGACACTTCCTGTCCGCTTTCACG obtaining a DNA of 216 pb that was 5’-labeled with [γ-^32^P]ATP and T4 polynucleotide kinase and used to analyze the interaction of ϕ29 SSB wild-type and mutants. The incubation mixture contained, in a final volume of 20 μl, 12 mM Tris-HCl, pH 7.5, 10 mM MgCl_2_, 1 mM dithiothreitol (DTT), 4% (v/v) glycerol, 0.1 mg/ml BSA, 3 nM of the labeled DNA heat denatured (5 minutes at 95°C), and 15, 30, 60 and 120 μM of ϕ29 SSB wild-type or mutants. After incubation for 15 minutes at 4 ^o^C, the samples were subjected to electrophoresis in precooled 4% (w/v) polyacrylamide gel [80:1 acrylamide/bis-acrylamide (w/w)] containing 12 mM Tris-acetate, pH 7.5, and 1 mM EDTA, and run at 4°C in the same buffer at 8 V/cm [32]. After autoradiography, ϕ29 SSB/DNA stable interaction was detected as a shift in the migrating position of the labeled DNA. Quantification of the percentage of DNA binding displayed by ϕ29 SSB wild-type and mutants was carried out by densitometry of the retarded band.

### ϕ29 TP-DNA amplification assay

The assay was performed essentially as described [25]. The incubation mixture contained, in 25 μl, 50 mM Tris-HCl, pH 7.5, 10 mM MgCl_2_, 20 mM ammonium sulfate, 1 mM DTT, 4% (v/v) glycerol, 0.1 mg/ml BSA, 80 μM each dNTP and [α-^32^P]dATP (1μCi), 30 pM of ϕ29 TP-DNA, 3 nM of ϕ29 DNA polymerase, 6.5 nM of ϕ29 TP, 35 μM of ϕ29 DBP, and 4, 8, 16 or 30 μM of wild-type or mutant SSB. After incubation for the indicated times, the reaction was stopped by adding 10 mM EDTA-0.1% SDS, and the samples were filtered through Sephadex G-50 spin columns. Quantitation of the DNA synthesized in vitro was carried out from the amount of radioactivity (Cerenkov radiation) corresponding to the excluded volume. The labeled DNA was denatured by treatment with 0.7 M NaOH and subjected to electrophoresis in alkaline 0.7% agarose gels [33] and then the gels were dried and autoradiographed.

### TP-DNA replication with an exonuclease-deficient ϕ29 DNA polymerase

The incubation mixture contained, in 25 μl, 50 mM Tris-HCl, pH 7.5, 10 mM MgCl_2_, 20 mM ammonium sulfate, 1 mM DTT, 4% (v/v) glycerol, 0.1 mg/ml BSA, 20 μM each dNTP and [α-^32^P]dATP (1μCi), 1.6 mM of ϕ29 TP-DNA, 13 nM of ϕ29 DNA polymerase (wild-type or exo- mutant D12A/D66A), 13 nM of ϕ29 TP, and 30 μM of either ϕ29 SSB wild-type or mutants. After incubation for 30, 60 and 90 minutes at 30°C, the reaction was stopped by adding 10 mM EDTA-0.1% SDS, and the samples were filtered through Sephadex G-50 spin columns. Quantitation of the DNA synthesized in vitro was carried out from the amount of radioactivity (Cerenkov radiation) corresponding to the excluded volume. The labeled DNA was denatured by treatment with 0.7 M NaOH and subjected to electrophoresis in alkaline 0.7% agarose gels [33] and then the gels were dried and autoradiographed.

### Strand displacement coupled to M13-DNA replication

The incubation mixture contained, in 25 μl, 50 mM Tris-HCl, pH 7.5, 10 mM MgCl_2_, 1 mM DTT, 4% (v/v) glycerol, 0.1 mg/ml BSA, 40 μM each dNTP and [α-^32^P]dATP (1μCi), 5 nM of primed M13mp18 ssDNA, 60 nM of ϕ29 DNA polymerase, and 30 μM of ϕ29 SSB wild-type or mutants. After incubation for 5 and 40 minutes at 30 ^o^C, the reaction was stopped by adding 10 mM EDTA-0.1% SDS, and the samples were filtered through Sephadex G-50 spin columns. Quantitation of the DNA synthesized *in vitro* was carried out from the amount of radioactivity (Cerenkov radiation) corresponding to the excluded volume. The labeled DNA was denatured by treatment with 0.7 M NaOH and subjected to electrophoresis in alkaline 0.7% agarose gels [33] and then the gels were dried and autoradiographed. The primed M13mp18 was obtained through hybridization of M13-DNA with the oligonucleotide 17mer at 70°C for 5 minutes. The mixture was slowly cooled to room temperature.

### Unwinding assay

The assay was performed essentially as described [22]. The incubation mixture contained, in 12.5 μl, 50 mM Tris-HCl, pH 7.5, 4% (v/v) glycerol, 0.1 mg/ml BSA, 2 nM labeled primed M13mp18 ssDNA, and 10, 20, 40 and 80 μM of ϕ29 SSB wild-type or mutants. After incubation for 60 minutes at 37 ^o^C, reactions were stopped with 1.25 μl of 0.25% (w/v) bromophenol blue, 0.25% (w/v) xylene cyanol, 30% (v/v) glycerol and 0.5% SDS as described [23]. The samples were subjected to electrophoresis at 4 ^o^C in 8% polyacrylamide gel containing 0.1% SDS. The gel was dried and autoradiographed. The substrate was obtained through hybridization of M13-DNA with the 5’-labeled oligonucleotide 17mer at 60°C for 5 minutes and slowly cooled to room temperature. Quantification of the percentage of displacement of the oligonucleotide by ϕ29 SSB wild-type and mutants was carried out by densitometry of the 5’-labeled 17 mer band resulting for the displacement of the oligonucleotide.

### Quantifications and statistics

All quantifications are indicated as the mean ± S.D. Statistical analyses were performed using Student’s t test.

## Results and discussion

### Role of residues Tyr50 and Tyr57 in ssDNA binding

The SSBs are structurally divergent, although most of them are predicted to contain an OB (oligonucleotide/oligosaccharide binding)-fold to bind ssDNA in a sequence-independent manner (reviewed in [13]). The structure of ϕ29 SSB is unknown and previous work was focused in the study of the binding characteristics by fluorescence spectroscopy. Previous studies showed that about 95% of the intrinsic tyrosine fluorescence of ϕ29 SSB is quenched upon binding to ssDNA, thus these tyrosines (Tyr50, Tyr57 and Tyr76) could be directly involved in complex formation with ssDNA [26, 27]. Therefore, we obtained mutants Y50A, Y57A and Y76A. The experiments were carried out with mutants Y50A and Y57A because variant Y76A was insoluble and could not be purified.

**Fig 1:**
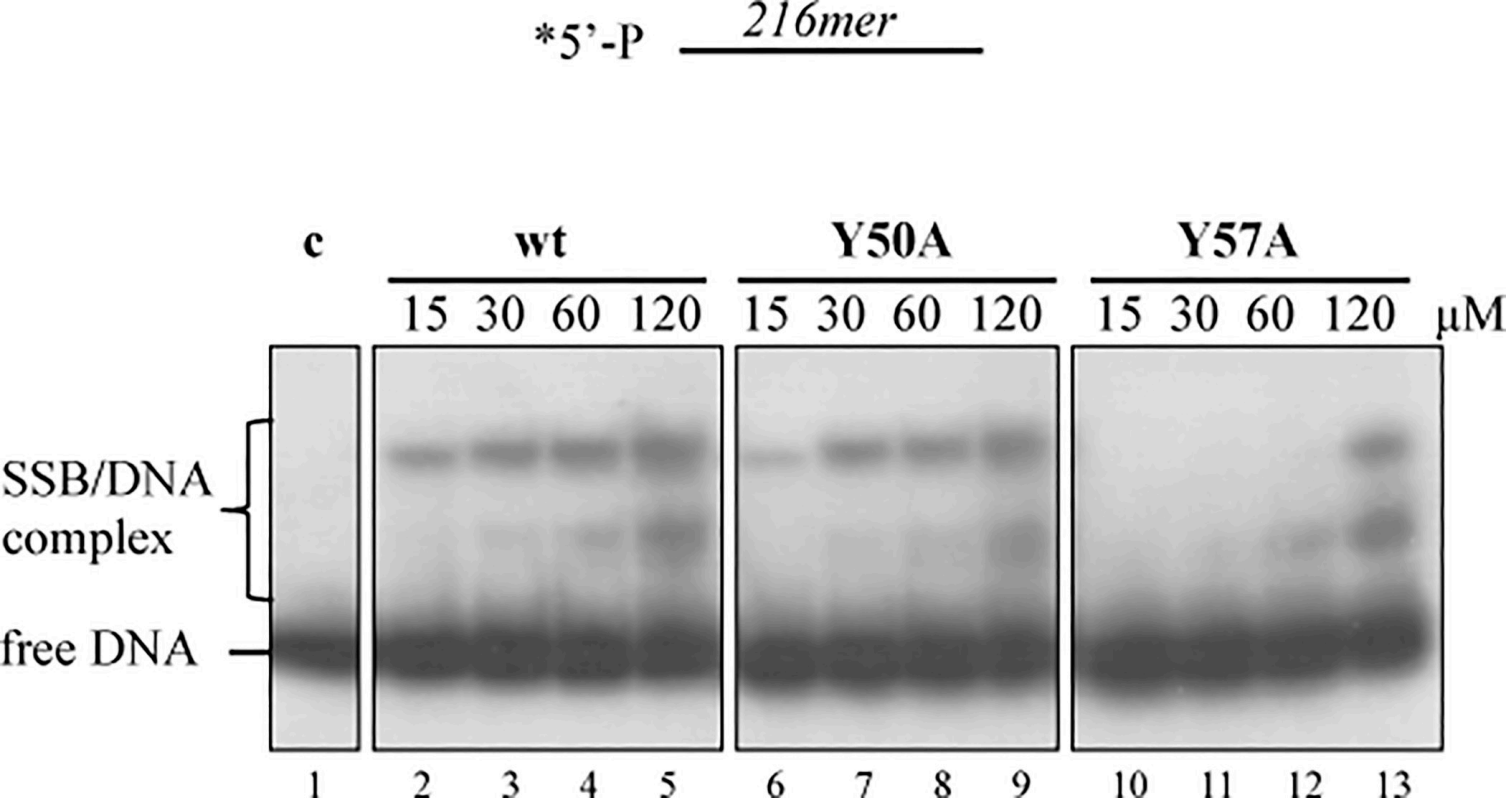
Gel mobility shift assay of the wild-type and mutants SSB. A 5’-labeled DNA fragment heat denatured (216mer) was incubated for 15 minutes with the indicated amounts of ϕ29 SSB wild-type or mutant at 4°C and subjected to non-denaturing gel electrophoresis. The bands corresponding to free DNA and to the SSB/DNA complex were detected by autoradiography. c: control without SSB. * asterisk indicates 5’ -labeled DNA fragment heat denatured (216mer).

To test if the SSB mutants were able to bind the ssDNA, we performed DNA mobility shift assays. For this purpose, a 5’-labeled DNA fragment heat denatured (216mer) was incubated with increasing amounts of ϕ29 SSB wild-type or mutants. As shown in Fig 1, the wild-type and mutant Y50A were able to produce two retarded bands, which are interpreted as a complex between ϕ29 SSB and ssDNA, being 96 ± 3% the percentage of binding displayed by mutant Y50A at 60 μM (Fig 1, line 8), respect to the wild-type SSB (Fig 1, line 4). However, mutant Y57A showed the more retarded band at the highest concentration only, displaying a 17 **±** 6% percentage of binding at 60 μM (Fig 1, line 12) (p<0.05 (t-test)), indicating a role of residue Tyr57 in DNA binding.

### TP-DNA amplification with the ϕ29 SSB variant Y57A was impaired

ϕ29 DNA replication starts at both origins of the viral genome by a protein priming mechanism [3]. The ϕ29 DNA polymerase catalyses the template-directed insertion of 5’ dAMP onto the viral TP and the subsequent processive DNA elongation coupled to strand displacement to produce full-length ϕ29 TP-DNA. A minimal replication system based on ϕ29 TP-DNA, TP and DNA polymerase can be used *in vitro* [6]. The addition to the reaction of DBP and SSB allows one thousand fold amplification of very few amount of initial ϕ29 TP-DNA [25].

**Fig 2:**
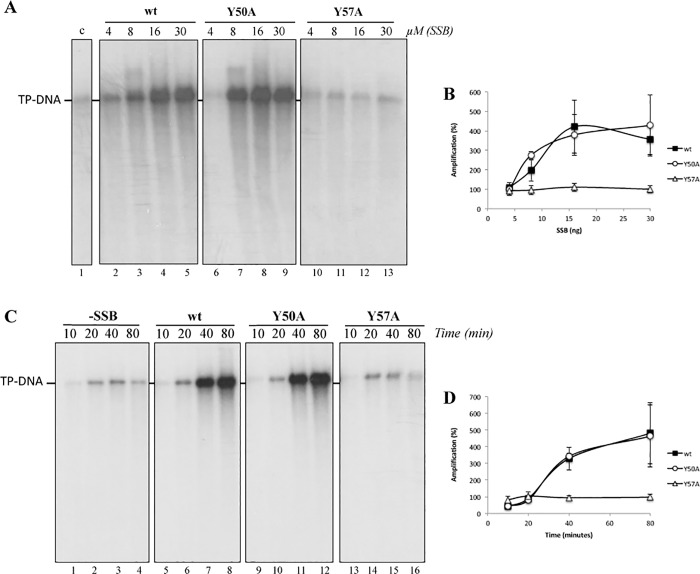
ϕ29 TP-DNA amplification. The assays were carried out as described in Materials and Methods using 3 nM of ϕ29 DNA polymerase, 30 pM of ϕ29 TP-DNA, 6.5 nM of ϕ29 TP, 35 μM of ϕ29 DBP binding protein and (A) increasing amounts of each ϕ29 SSB or (C) 30 μM of each ϕ29 SSB at different times. The reactions were incubated at 30°C for 80 minutes in A and for the indicated times in B. The length and amount of the synthetized DNA was analyzed by 0.7% alkaline agarose gel electrophoresis followed by autoradiography. c: control without SSB (Fig 2A, line 1). Percentage of amplification obtained in A and C (respect to the control without SSB) are represented in B and D, respectively, as mean **±** SD corresponding to three independent experiments: wt SSB (black squares), mutant Y50A (white) mutant Y57A (white triangles).

The ability of the ϕ29 SSB mutants to stimulate viral DNA replication was analyzed in TP-DNA amplification assays. First, we carried out the assay in the presence of increasing amounts of ϕ29 SSB (Fig 2A and 2B). The results showed that mutant Y50A (Fig 2A, lines 6–9) displayed essentially a wild-type behaviour (lines 2–5; see also Fig 2B) not showing statistically significant differences (t-test), whereas mutant Y57A (lines 10–13) was severely impaired (p<0.05 when used 8, 16 and 30 μM SSB (t-test)). In addition, we performed amplification kinetic assays to compare the effect of the mutants and the wild-type ϕ29 SSBs at different times. As shown in Fig 2C and 2D, the efficiency displayed by mutant Y50A (Fig 2C, lines 9–12) was similar to the wild-type ϕ29 SSB (lines 5–8; see also Fig 2D), not showing statistically significant differences (t-test). On the other hand, mutant Y57A (lines 13–16) was unable to stimulate the TP-DNA amplification, showing a replication amount comparable with the one obtained without adding SSB (lines 1–4) (p<0.05 at 40 and 80 minutes (t-test)).

### Involvement of ϕ29 SSB residue Tyr57 in strand displacement

As mentioned before, ϕ29 DNA polymerase is able to couple processive DNA synthesis to strand displacement in the absence of accessory proteins. The minimal ϕ29 replication system, *in vitro*, is based on the ϕ29 TP-DNA, TP and DNA polymerase [6]. Despite the fact that ϕ29 SSB does not display any stimulatory effect in the early replication step, it has been shown to stimulate the elongation rate during strand displacement DNA replication [22]. The ϕ29 SSB binds to ssDNA with relative low affinity and moderate cooperativity, covering 3–4 nucleotides per monomer [26].

**Fig 3:**
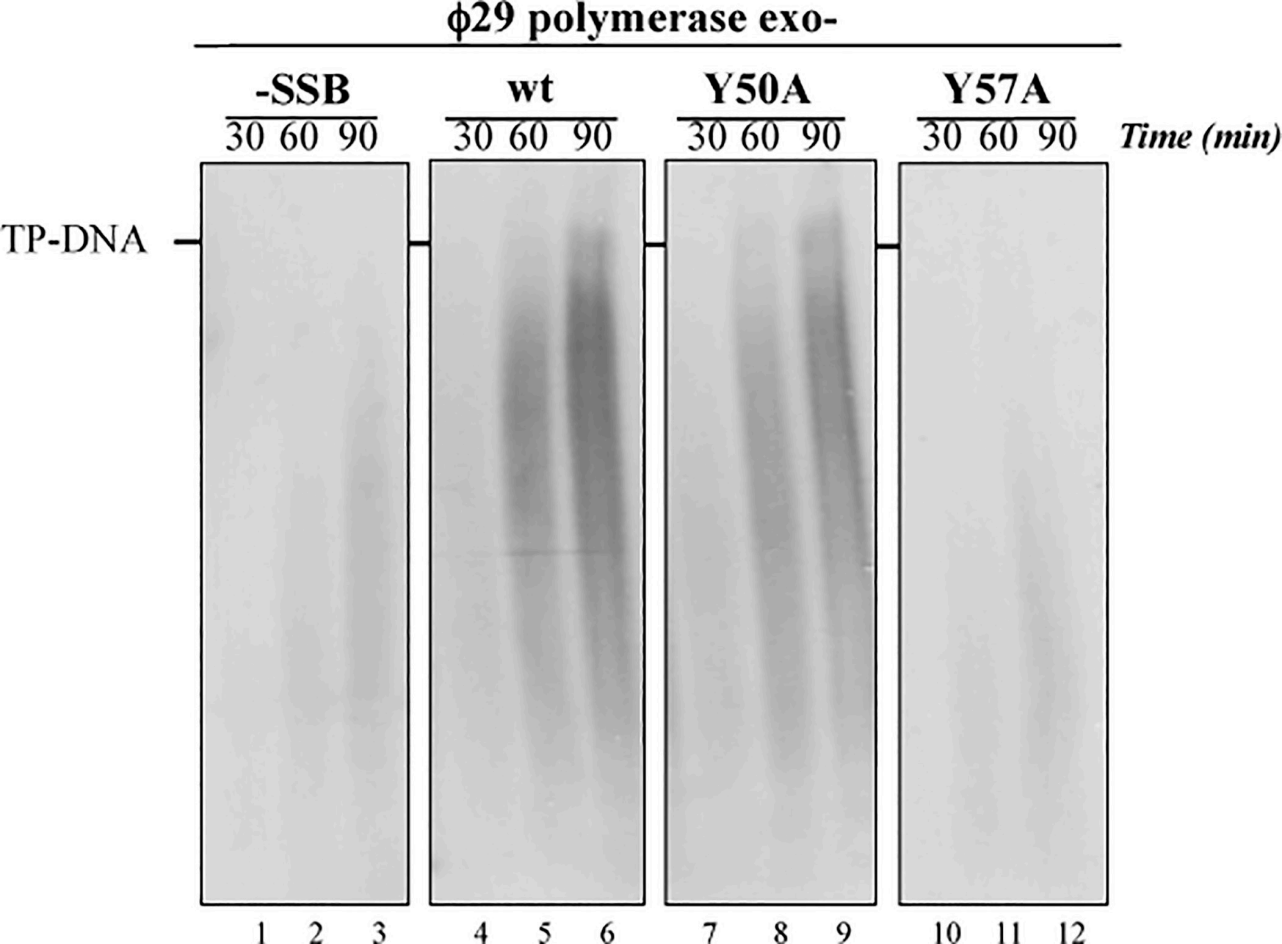
TP-DNA replication with the exonuclease-deficient ϕ29 DNA polymerase mutant. The assay was carried out as described in Materials and Methods in the presence of 13 nM of ϕ29 DNA polymerase exo- mutant D12A/D66A, 1.6 nM of TP-DNA, 13 nM of TP and 30 μM of SSB. The samples were incubated at 30°C for the indicated times. The size of the replication products was analyzed by 0.7% alkaline agarose gel electrophoresis followed by autoradiography. The position of unit-length TP-DNA is indicated.

We analyzed the effect of ϕ29 SSB mutants on the DNA elongation rate under conditions in which strand opening is impaired. For that we used the exonuclease-deficient ϕ29 DNA polymerase variant D12A/D66A with two of the catalytic aspartic acids mutated into alanine [29] that is also impaired in strand displacement capacity [34, 35]. When the SSB wild-type (Fig 3, lines 4–6) or mutant Y50A (Fig 3, lines 7–9) were added to the reaction we could see a stimulatory effect in the rate and amount of DNA elongation. However, the replication carried out without SSB (Fig 3, lines 1–3) and with mutant Y57A (Fig 3, lines 10–12) were similar, being this mutant unable to stimulate the DNA elongation.

**Fig 4:**
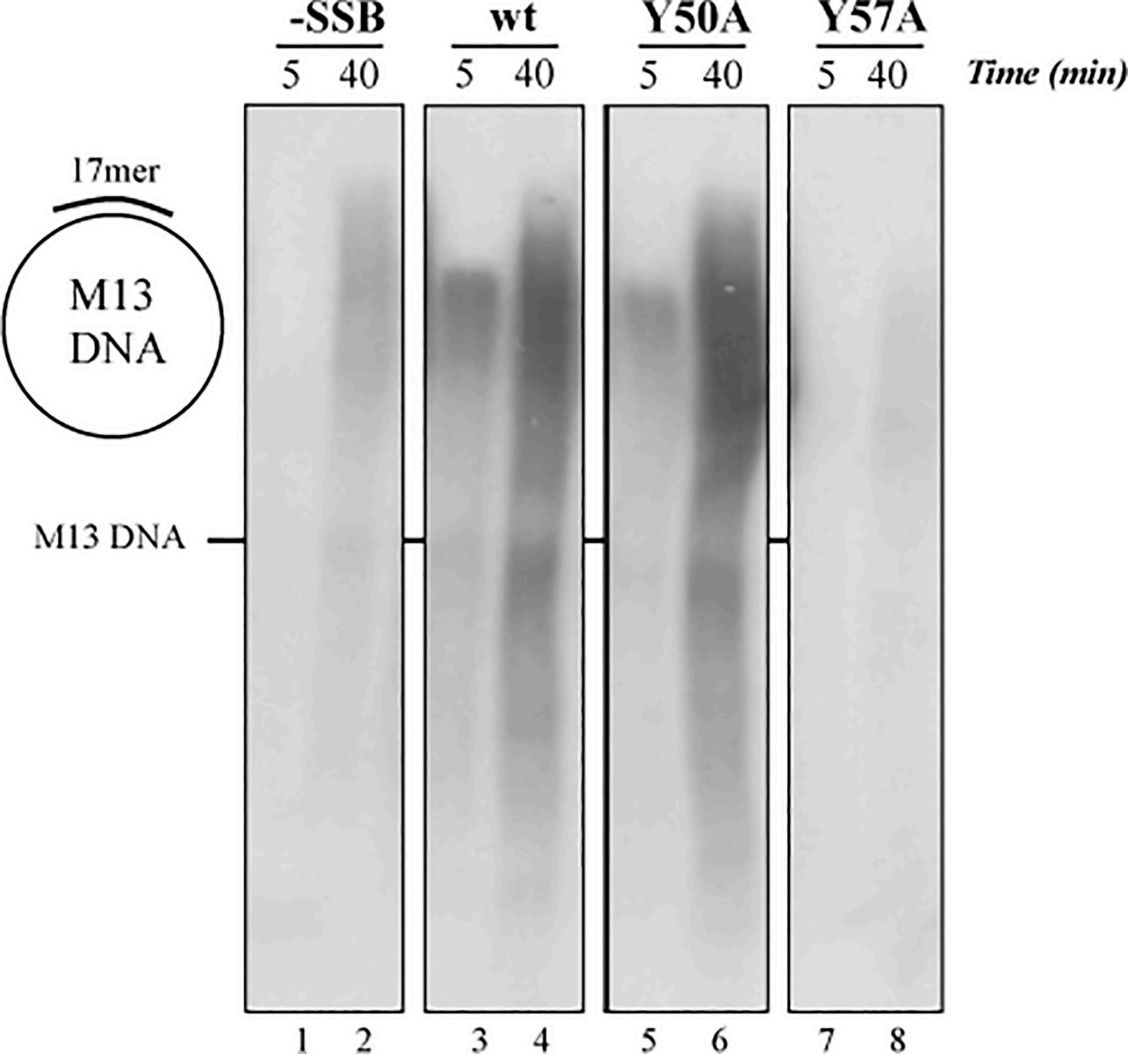
Primed M13 ssDNA replication. The assay was performed as described in Materials and Methods using 60 nM of ϕ29 DNA polymerase, 5 nM of M13-17mer and 30 μM of the different SSBs. Samples were incubated at 30°C for the indicated times and analyzed by 0.7% alkaline agarose gel electrophoresis followed by autoradiography. The position of unit-length M13 DNA is indicated.

We also studied the effect of the SSB mutants on DNA elongation using as substrate primed M13 DNA (M13 DNA hybridized to a 17mer oligonucleotide as described in Materials and Methods). We performed a singly-primed M13 DNA replication assay, in which ϕ29 DNA polymerase performs primer elongation using the 3’-OH group of the 17mer. The first replication round does not require strand displacement, but once this round is completed, upon reaching the 5’ terminus of the primer oligonucleotide, it is necessary the coupling of polymerization and strand displacement to continue DNA synthesis. One of the main characteristics of the ϕ29 DNA polymerase is the strand displacement capacity and, as we described above, the ϕ29 SSB has the capacity to stimulate the strand displacement during the DNA replication. ϕ29 DNA polymerase was able to carry out the first round and the next rounds of replication without accessory proteins and, in the presence of ϕ29 SSB wild-type (Fig 4, lines 3 and 4) and mutant Y50A (Fig 4, lines 5 and 6), replication was strongly stimulated at the times assayed. However, mutant Y57A (Fig 4, lines 7 and 8) was unable to stimulate the M13 DNA replication, according with the results of the TP-DNA replication assay.

### Unwinding activity of ϕ29 SSB

The genomes of cellular organisms are organized as dsDNA which contain all the genetic information. For the use of this information, the double helix DNA must be unwound. DNA unwinding has risks, because the ssDNA can form secondary structures or be attacked by nucleases. SSBs solve these problems binding ssDNA and are involved in processes as DNA replication, recombination, repair and replication restart (reviewed in [11, 12]).

**Fig 5:**
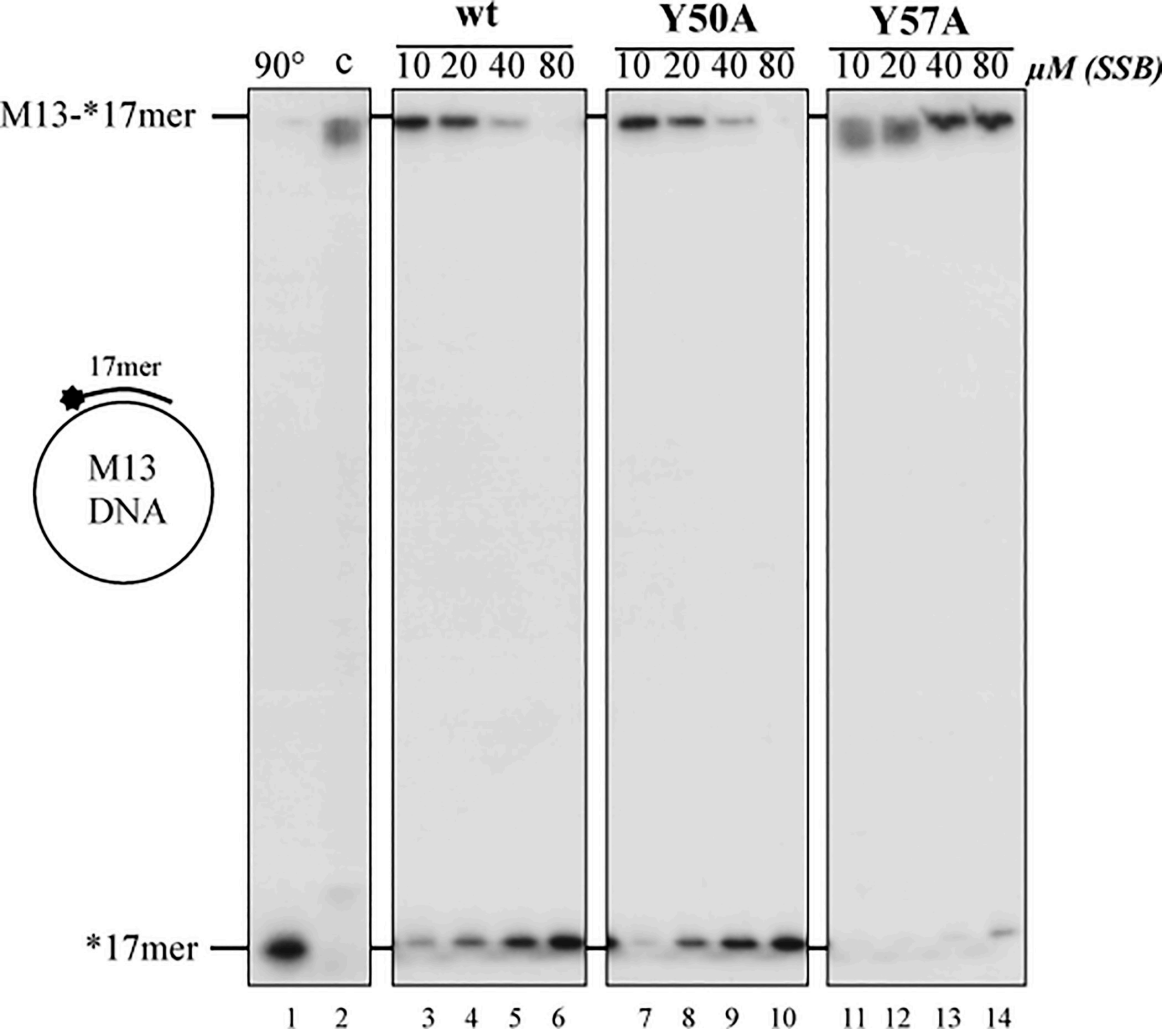
Helix destabilizing activity of ϕ29 SSB wild-type and mutants. The assay was carried out as described in Materials and Methods using 2 nM of M13-17mer and increasing amounts of each SSB being incubated at 37°C for 60 minutes. The samples were fractionated in an 8% polyacrylamide gel followed by autoradiography. 90°C: heat-denatured substrate; c: without SSB. *asterisk indicates 5’ -labeled oligonucleotide 17mer.

ϕ29 SSB has helix-destabilizing activity and is able to displace oligonucleotides annealed to M13 ssDNA. This activity is independent of energy, corresponding to a DNA unwinding rather than to a helicase activity [22]. To study the unwinding ability of the SSB mutants, we performed the unwinding assay using as substrate a full-length M13 ssDNA molecule hybridized to a 5’-labeled 17mer oligonucleotide. This substrate was incubated with increasing amounts of SSB. ϕ29 SSB wild-type was able to unwind the 5’-labeled 17mer from the M13 DNA resulting in a full displacement of the 17mer oligonucleotide, as shown in Fig 5 (lines 3–6). Mutant Y50A behaved in a wild-type fashion (lines 7–10); however, mutant Y57A was clearly impaired, and even at the highest amount of protein assayed there was very little displacement of the 17mer oligonucleotide, with a percentage of displacement of the oligonucleotide respect to the wild-type of 17 ± 6% (p<0.05 (t-test)), in contrast with 97 ± 3% displayed by mutant Y50A at the same amount of protein assayed (80 μM, see lines 14 and 10 in Fig 5). As a control, we carried out the assay in the absence of ϕ29 SSB and did not result in release of the labeled oligonucleotide (line 2), indicating that the hybrid substrate was stable. In contrast, when the temperature was raised up to 90°C, there was a complete displacement of the 17mer oligonucleotide (line 1), as expected. This impaired helix destabilizing activity displayed by mutant Y57A explains the results obtained in the TP-DNA replication with the exonuclease–deficient DNA polymerase (Fig 3) and in the primed M13 ssDNA replication (Fig 4) where the mutant was unable to stimulate the DNA elongation.

Altogether, the above results indicate that mutant Y50A was able to form a complex with ssDNA and displayed a wild-type helix-destabilizing activity, stimulating the elongation of replication in the substrates assayed. Therefore, residue Tyr50 does not seem to play an essential role in the SSB activity. Conversely, mutant Y57A did not stimulate viral DNA replication in TP-DNA amplification, probably due to its impaired ssDNA binding. Moreover, mutant Y57A was unable to efficiently displace the labeled oligonucleotide from M13 ssDNA, resulting in a lack of stimulation of the elongation rate during strand displacement DNA replication using as substrates TP-DNA or M13-DNA. Thus, residue Tyr57 plays an important role in the activity of ϕ29 SSB due to the involvement of this residue in binding the ssDNA. The lack of this interaction is affecting the activities of the SSB being the derivative mutant (Y57A) unable to unwind the DNA or to stimulate the DNA elongation.

## Supporting information

S1 Fig Induction and purification of ϕ29 SSB. To the left the expression tests with the total (T) and soluble (S) proteins without induction (-I) and total and soluble proteins after induction (+I) with IPTG as indicated in Materials and Methods. To the right, the main steps of purification are indicated as: Lys: lysate; SHV: super high velocity; SPEI: super polyethylenimine; PAS 30%: pellet AS 30%; Eluted PH/Q columns: eluted from phosphocellulose and mono Q column (1, 2 and 3 were eluted at 50 mM NaCl and 4 was eluted at 75 mM NaCl); PAS 65%: pellet AS 65%, +D: after dialysis, M: marker (SSB purified as described [22]). The samples were analyzed by 15% polyacrylamide gel electrophoresis.
(TIF)

S2 Fig Electrophoresis of purified ϕ29 SSB wild-type and mutants. Aliquots (500 ng) of the purified preparations of wild-type SSB and the indicated mutants were analyzed in 15% SDS/PAGE. Polypeptides were visualized by staining the gel with Coomassie blue dye. The positions and size (in kDA) of the marker polypeptides (c) (New England Biolabs) are indicated on the left.
(TIF)

S1 Text Protein purification. (DOCX)

S2 Text Protocol for the purification of the ssM13mp18 DNA substrate. (DOCX)
