# Supplementary figures and images for: Tyrosines involved in the activity of φ29 single-stranded DNA binding protein
Source: PLoS One. 2019 May 20;14(5):e0217248. doi: 10.1371/journal.pone.0217248 (PMC6527236; doi:10.1371/journal.pone.0217248)

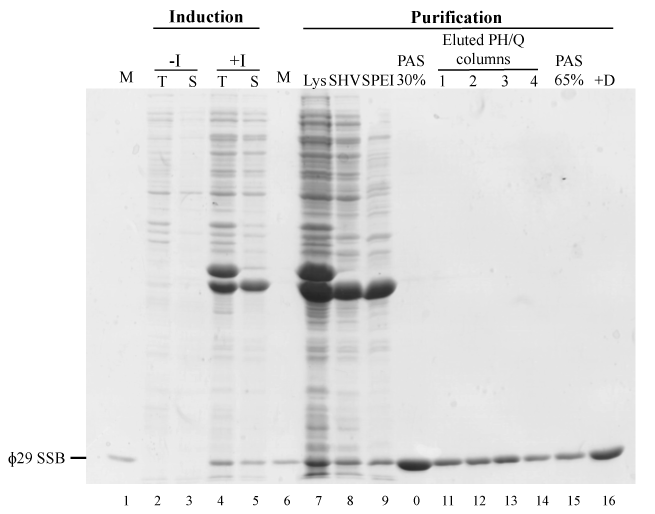

Supplement: S1 Fig — To the left the expression tests with the total (T) and soluble (S) proteins without induction (-I) and total and soluble proteins after induction (+I) with IPTG as indicated in Materials and Methods. To the right, the main steps of purification are indicated as: Lys: lysate; SHV: super high velocity; SPEI: super polyethylenimine; PAS 30%: pellet AS 30%; Eluted PH/Q columns: eluted from phosphocellulose and mono Q column (1, 2 and 3 were eluted at 50 mM NaCl and 4 was eluted at 75 mM NaCl); PAS 65%: pellet AS 65%, +D: after dialysis, M: marker (SSB purified as described [22]). The samples were analyzed by 15% polyacrylamide gel electrophoresis. (TIF) [file pone.0217248.s001.tif]

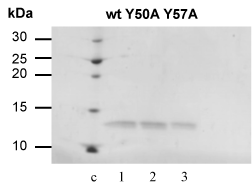

Supplement: S2 Fig — Aliquots (500 ng) of the purified preparations of wild-type SSB and the indicated mutants were analyzed in 15% SDS/PAGE. Polypeptides were visualized by staining the gel with Coomassie blue dye. The positions and size (in kDA) of the marker polypeptides (c) (New England Biolabs) are indicated on the left. (TIF) [file pone.0217248.s002.tif]
