# Supplementary material for: Tyrosines involved in the activity of φ29 single-stranded DNA binding protein
Source: PLoS One. 2019 May 20;14(5):e0217248. doi: 10.1371/journal.pone.0217248 (PMC6527236; doi:10.1371/journal.pone.0217248)
Supplement: S1 Text — (DOCX) [file pone.0217248.s003.docx]

**S1 Text. Protein purification:** SSB mutants Y50A, Y57A and Y76A were obtained using the QuickChange Site-Directed Mutagenesis Kit provided by Stratagene, using as template the plasmid pT7-3 containing the viral gene 5 that encodes the wild-type φ29 SSB. The presence of the desired mutations, as well as the absence of additional ones, was determined by sequencing the entire gene. The wild-type and mutant plasmids were overexpressed in XL1-Blue *Escherichia coli* cells and, then, purified.

*E. coli* Solub-BL21 (DE3) strain was transformed with plasmid pT7-3 containing the gene 5 wild-type or mutants. The cells were grown at 30 ^o^C in LB broth in the presence of ampicillin up to an optical density of 0.6. After 45 minutes, the temperature was raised to 37 ^o^C and the expression of the φ29 SSBs was induced with 0.5 mM IPTG for 4 hours and 30 minutes. Cultures were centrifuged 11 minutes at 4 ^o^C at 5,000 rpm in a GSA rotor to remove the culture medium. Part of the pellet was suspended in buffer 6 (50 mM Tris-HCl, pH 7.5, 5% (v/v) glycerol, 7 mM β-mercaptoethanol, 1 mM EDTA) supplemented with 0.2 M NaCl and sonicated to lyse the cells. Part of the lysate was centrifuged at 4 ^o^C for 20 minutes at 20370 g to analyze the soluble protein in the supernatant.

Grown bacteria (14.6 grams) were pelleted and lysed with 17.5 g of alumina. The lysates were resuspended in buffer 6 supplemented with 0.2 M NaCl. To remove the alumina, the lysates were centrifuged 5 minutes at 2,000 rpm at 4 ºC in a GSA rotor and the supernatants were recollected (lysate). The cleared lysates were centrifugated again 20 minutes at 12,000 rpm at 4 ºC in a GSA rotor to separate the soluble fraction (super high velocity). The DNA in the soluble extract was precipitated with 10% polyethylenimine and centrifugated at 4 ºC for 10 minutes at 12,000 rpm in a GSA rotor. The SSBs were in the supernatant (super polyethylenimine). We precipitated them with ammonium sulphate (AS) to 35% saturation to obtain polyethylenimine-free protein after centrifugation during 30 minutes at 4 ºC at 12,000 rpm in a GSA rotor. These pellets were resuspended in buffer 6 supplemented with 30% AS and centrifuged 30 min at 4 ºC at 12,000 rpm in a GSA rotor (pellet ammonium sulphate 30%). The resulting pellets were resuspended in buffer 6 and passed through phosphocellulose and mono Q columns, previously equilibrated with the same buffer. The proteins were eluted first with buffer 6 supplemented with 50 mM NaCl, then with buffer 6 with 75 mM NaCl and, finally, with buffer 6 with 0.1 M NaCl (eluted PH/Q columns). The eluted fractions with the highest concentrations of proteins were joined, precipitated with AS 65% and centrifuged for 30 minutes at 4 ºC at 12,000 rpm in a GSA rotor (pellet ammonium sulphate 65%). These pellets were resuspended in buffer 6 with 0.5 M NaCl and dialysed in a Whisking membrane against buffer 6, 50% glycerol, 0.025% tween (after dialysis). S1 Fig shows the principal steps of the purification of the wild-type SSB. The same method was followed to purify the SSB mutants. The purified proteins are shown in S2 Fig.
