# Supplementary material for: Tyrosines involved in the activity of φ29 single-stranded DNA binding protein
Source: PLoS One. 2019 May 20;14(5):e0217248. doi: 10.1371/journal.pone.0217248 (PMC6527236; doi:10.1371/journal.pone.0217248)
Supplement: S2 Text — (DOCX) [file pone.0217248.s004.docx]

**S2 Text. Protocol for the purification of the ssM13mp18 DNA substrate:**

Isolation of M13 phage: Grow 10 ml of XL1Blue Escherichia coli cells and infect with M13 (at a MOI of 100) during 30 minutes at 37 ºC. Add LB medium until 1 litre and incubate during the night at 37 ºC. Centrifuge 10 minutes at 8000 rpm in a GS3 rotor at 4 ºC. Collect the supernatant and add 5 volumes of 20% polyethylene glycol (PEG) 6000 and sodium chloride at 2.5 M. Incubate 2 hours at 4 ºC and centrifuge 15 minutes at 12000 rpm in a GSA rotor at 4 ºC. Resuspend the pellet with 10 mM Tris-HCl, pH 7.5 and 1mM EDTA and centrifuge 10 minutes at 10000 rpm in a SS34 rotor. Incubate the supernatant with 10 mM magnesium chloride and DNase and RNase (10 µg/ml) during 1 hour at 37 ºC and then 10 minutes at 65 ºC. Precipitate with 5 volumes of 20% polyethylene glycol (PEG) 6000 and sodium chloride at 2.5 M and centrifuge 15 minutes at 12000 rpm in a SS34 rotor at 4 ºC. Resuspend the pellet with 20 ml of phage diluent (50 mM Tris-HCl, pH 7.8, 0.1 M sodium chloride and 10 mM magnesium chloride). Centrifuge 20 minutes at 13000 rpm in a SS34 rotor and resuspend the pellet with phage diluent. Add cesium chloride (1.31 g/ml) and centrifuge 24 hours at 45000 rpm in a T865 rotor at room temperature. Collect the phage band of the gradient and dialyse it against phage diluent.

Isolation of single-stranded DNA from M13 phage: Incubate the isolated phage with 10% SDS and proteinase K (75µg/ml) during 3 hours at 37 ºC. Add 1 volume of phenol and invert to mix. Centrifuge 10 min at 10000 rpm in a SS34 rotor at room temperature. Add to the aqueous solution 1 volume of phenol/ chloroform and centrifuge in the same conditions (repeat two times). Select the aqueous solution and add 1 volume of ether and mix. Remove the top part of the solution and add again 1 volume of ether. Remove the top part. Add sodium acetate to 0.3 M and 5 volumes of ethanol. Incubate 2 hours at -70 ºC. Centrifuge 20 minutes at 13000 rpm in a SS34 rotor. Remove the supernatant and wash the pellet with 80% ethanol. Air-dry during the night and resuspend the pellet in 1.5 ml of 10 mM Tris-HCl, pH 7.5 and 1mM EDTA at 4 ºC.
